# Multiscale Entropy-Based Feature Extraction for the Detection of Instability Inception in Axial Compressors

**DOI:** 10.3390/e26010048

**Published:** 2024-01-02

**Authors:** Yihan Fu, Zheng Zhao, Peng Lin

**Affiliations:** School of Automation, Hangzhou Dianzi University, Hangzhou 310018, China; 22060419@hdu.edu.cn (Y.F.); 222060186@hdu.edu.cn (Z.Z.)

**Keywords:** multiscale entropy, nonlinear feature, short time series, instability inception, axial compressors

## Abstract

The detection of instability inception is favorable to avoid compressor instability. In this paper, a multiscale entropy-based feature extraction is developed for the detection of the instability inception in axial compressors. Nonlinear and statistical features of the short-time instability inception are extracted by generally combining multiscale entropy and statistical features. First, nonlinear features are extracted by refined composite multiscale entropy to avoid the inaccurate estimation or undefined entropy of multiscale entropy for short time series. Second, the time-domain-based statistical features are chosen to capture more information on instability inception, and the dominant statistical features are determined by random forests implemented with the mean decrease accuracy algorithm at each time scale. The obtained refined composite dominant statistical features are regarded as weighting factors and integrated with the refined composite multiscale entropy to generate a combined feature. Finally, numerical simulation results on two synthetic noise datasets and a compressor instability model dataset are presented to demonstrate the effectiveness, efficiency, and robustness of the combined features under different conditions.

## 1. Introduction

Compressor instability, which usually includes rotating stall and surge, is the most serious phenomenon in compressor operation and severely limits the performance of axial compressors [1]. Rotating stall is a severely asymmetric distribution of axial velocity around the annulus of the compressor, which occurs as the breakdown of orderly flow through the blade passage and causes the pressure rise to drop dramatically. Surge is a large-amplitude limit-cycle oscillation of both pressure rise and mass flow through the entire compression system and causes possibly reversed flow or flame-out of the compressor [2]. Preventing instability is an important aspect of safety and stability in axial compressors. In the field of fault diagnosis, an effective approach is to early detect compressor instability by accurately identifying the instability inception. Therefore, it is of significance to identify instability inception before compressor instability.

The mechanism of compressor instability has been extensively researched through theories and experiments [2]. It is generally accepted that the flow separation in an individual blade results in the onset of compressor instability. A detailed explanation of the flow separation along the blade row was provided by Emmons et al. [3] and the basic compressor instability model derived by Moore and Greitzer [4] was proposed to describe the evolution of the compressor instability. With respect to experimental research, instability inception data could be measured using circumference-distributed pressure sensors on the compressor casing wall when the compressor was throttled into instability. Various analysis approaches such as spatial Fourier decomposition [5,6], the traveling wave energy [7], wavelet analysis [8,9,10], and correlation [11,12] were further developed to classify instability inception and understand the happen of instability. In researching instability models, a fundamental compressor instability model was developed as a nonlinear third-order ordinary differential equation. It characterizes the axial and circumferential unsteady flow field in a compressor, capturing crucial nonlinearities that influence instability inception in experiments [5]. Based on the basic model, numerous physical models were proposed to describe and control the nonlinear instability behavior [6,13,14].

By analyzing the statistical properties of the measured data on two axial compressors, Inoue et al. [15] observed that the periodicity of the pressure fluctuation gradually disappeared with the occurrence of compressor instability. Thus, the instability inception could be predicted by tracking the periodicity of the pressure fluctuation, the collapse of the periodicity could be considered to be the occurrence of compressor instability. Auto-correlation, cross-correlation [11,12], root mean square (RMS) [16] and $R_{C}$ parameter [17,18] have been investigated to track the periodicity by monitoring the irregularity or the dissimilarity in the pressure fluctuation. The performance of correlation and RMS approaches has been evaluated on an axial compressor in [19,20]. The effectiveness of these two approaches was highly dependent on practical aspects like rotational speed fluctuation, the location of the sensors, throttling processes, and intake air conditions. In addition, Young et al. [16] revealed that the increase in irregularity in the blade passing pressure fluctuations was dependent on tip-clearance size and eccentricity. Therefore, it is difficult to obtain reliable instability detection results by using only a single index.

For the nonlinear dynamic features of compressor instability, complexity measures in the underlying nonlinear dynamic process have been introduced as an alternative approach to identify and detect instability inception. The entropy-based approaches can extract the nonlinear features hidden in the obtained data and have been widely applied in the fault diagnosis of rotating machinery [21,22,23,24,25,26]. Ribeiro et al. [27] built the entropy universe by describing in-depth the relationship between the most applied entropies in time series for different scientific fields, establishing bases for researchers to properly choose the variant of entropy most suitable for data. Xing et al. [28] proposed an improved hierarchical multi-scale reserve dispersion entropy (HMRDE) method to analyze the frequency difference features of incipient fault signals. The HMRDE enhanced the disorder differences between each state signal and improved the distinguishing ability of classifier inputs, solving the problem of MRDE’s omission of obvious fault features in a higher frequency range and resulting in higher classification accuracy for the classifiers. Feng et al. [29] proposed the frequency-domain fuzzy-entropy algorithm to measure the frequency-domain complexity of the vibration signal and effectively extract the fault information contained in the vibration signal of the check valve. A novel health indicator was developed based on cyclic-correntropy to monitor the gear surface degradation induced by gear wear progression [30]. With the help of the indicator developed, the health status of the gearbox could be evaluated and serve as a valuable non-destructive monitoring tool for assessing the gear transmission system degradation status in industry practices. To obtain the reliability index of fault diagnosis, a combined feature derived from the multiple features fusion benefits from the advantage of a single feature to the fault diagnosis. A feature extraction approach SPmIMPE, which composes dominant statistical parameters and permutation entropy, was proposed to deal with the nonlinear and non-stationary nature of vibration signals [31]. The fault type and fault severity of the bearing could be simultaneously recognized over a wide range of operating conditions based on the SPmIMPE algorithm. In compressor instability analysis, a detailed procedure of approximate entropy (ApEn) algorithm was presented for the first time to identify the instability inception in axial compressors [32]. The amount of irregularity and unpredictability of pressure fluctuation was measured by ApEn and spikes in ApEn occurred before the compressor instability.

Nevertheless, the traditional entropy approach has two shortcomings in the measurement of the complexity of the system. On the one hand, the traditional entropy may fail to correctly quantify the complexity of the system because time series derived from the complex system are likely to present structures on multiple spatiotemporal scales [33]. On the other hand, a small amount of the time series will lead to inaccurate or undefined entropy in the calculation of the entropy because the entropy changes significantly as a function of the parameters’ subseries length, similarity tolerance, and data length. The first shortcoming can be improved by multiscale entropy, which quantifies the complexity of the system at different time scales. Costa et al. [33] proposed the multiscale entropy (MSE) algorithm to represent the complexity of a time series by calculating sample entropy (SampEn) over a range of time scales. The MSE algorithm resolves the contradiction between the lower entropy and higher complexity of 1/f noise compared with white noise. However, the coarse-graining procedure in the MSE algorithm reduce the number of data points and lead to inaccurate results in entropy calculation. Humeau et al. [34] reviewed several algorithms that have been introduced to improve the estimation of MSE by increasing the accuracy of the entropy estimates and exploring alternative coarse-graining procedures. In the case of short time series, a refined composite multiscale entropy (RCMSE) was proposed to compensate for the inaccurate calculation of entropy [35]. Simulation results in [35] revealed that the RCMSE algorithm could be used to increase the accuracy of entropy estimation and reduce the probability of inducing undefined entropy compared with the MSE algorithm. Azami et al. [36] investigated different alternatives to coarse-graining in complexity approaches and assessed the impact of coarse-graining in multiscale entropy estimations based on both Sample Entropy and Dispersion Entropy. Their results confirmed that the refined composite approach may improve the stability of entropy results when dealing with short or noisy signals.

Motivated by previous research [31,35], we propose a multiscale entropy-based feature extraction algorithm, abbreviated as wRCMSE, to identify the compressor instability inception. To improve the reliability of instability detection from a single index, a combined feature is constructed by integrating the dominant refined composite statistical features (RCSFs) and the RCMSE. As compressor instability is a constantly evolving nonlinear dynamic process, nonlinear dynamic changes can be detected by the RCMSE, which quantifies the degree of complexity of the short-term instability inception over a range of time scales. Based on a refined composite coarse-grained procedure, the multiscale time-domain-based statistical features are derived to capture more information on instability inception, and RCSFs are determined by a random forest implemented mean decrease accuracy algorithm. Furthermore, RCSFs can be regarded as weighting factors and multiplied (element-wise) with the RCMSE at each scale to generate the wRCMSE. The main contributions of this paper are as follows: (1) We propose a multiscale entropy-based feature extraction algorithm to identify the short-time instability inception in axial compressors. This algorithm effectively captures the changes in the system behavior across multiple time scales. (2) By integrating multiscale RCSFs and multiscale entropy, we derive a combined feature that provides a more comprehensive representation of system instability. This combined feature leverages the complementary information from both entropy and RCSFs, enhancing the identification accuracy.

The rest of this paper is organized as follows. The established entropy methods are described in Section 2. The main results are given in Section 3, including the extraction of RCSFs via random forests and the formation of wRCMSE. Section 4 shows some numerical simulation results, and Section 5 gives the conclusion of this paper.

## 2. Description of the Established Entropy

In this section, we briefly introduce the theoretical backgrounds of the SampEn and the RCMSE algorithm.

### 2.1. The SampEn Algorithm

SampEn was introduced by Richman et al. [37] as a model-independent quantification of the complexity of a time series. The complexity of a time series is estimated using SampEn by measuring the probability of generating new patterns in the time series. If the SampEn value is greater, then the greater will be the value of the complexity of the time series.

Let $X=\{x_{1},\cdots ,x_{N}\}$ represent a time series of length *N*; the SampEn algorithm mainly includes the following three aspects:

1. Phase space reconstruction, the original vector *X* can be reconstructed in terms of the phase space vectors: $X_{i}^{m}=\left\{x_{i},\cdots ,x_{i+m-1}\right\}$ with 1≤i<N−m+1, where *m* is the embedding dimension.
2. A similarity measure, the distance of vectors $X_{i}^{m}$ and $X_{j}^{m}$, $d[X_{i}^{m},\;X_{j}^{m}]$, is the maximum absolute difference in the scalar components $\underset{k=1,\cdots ,m}{max}\left(X_{i}^{m}\left(k\right)-X_{j}^{m}\left(k\right)\right)$. For a given tolerance *r*, two similar vectors $X_{i}^{m}$ and $X_{j}^{m}$ are defined as matched vector pairs if $d[X_{i}^{m},\;X_{j}^{m}]<r,\;i\neq j$.
3. Calculate SampEn, the negative natural logarithm of the empirical probability that $d[X_{i}^{m+1},\;X_{j}^{m+1}]<r$ given that $d[X_{i}^{m},\;X_{j}^{m}]<r$, $SampEn\left(m,r,N\right)=-ln\left(\frac{B^{m+1}\left(r\right)}{B^{m}\left(r\right)}\right)$ where $B^{m}\left(r\right)=\frac{1}{N-m}\sum_{i=1}^{N-m}\left(\frac{1}{N-m-1}n_{i}^{m}\right)$, $n_{i}^{m}$ represents the count of matched vector pairs with $X_{i}^{m}$.

The embedding dimension *m* and the tolerance *r* are two important parameters in the calculation of SampEn. m=2 and r=0.4σ, where σ is the standard deviation of the time series, were suggested for ApEn in the analysis of rotating machinery [38].

### 2.2. The RCMSE Algorithm

The RCMSE algorithm has been proposed by Wu et al. [35] to overcome the variance of estimated entropy values at large scales and undefined entropy values for short time series. The RCMSE algorithm is composed of two steps as follows:

1. For a given scale factor τ, the original time series *X* is divided into non-overlapping windows of length τ and the data points inside each window are averaged. The *k*th (k=1,2,⋯,τ) coarse-grained time series $y_{k}^{\left(\tau \right)}=\left\{y_{k,1}^{\left(\tau \right)},y_{k,2}^{\left(\tau \right)},\cdots ,y_{k,p}^{\left(\tau \right)}\right\}$ is defined as [35]
  (1) $$y_{k,j}^{\left(\tau \right)}=\frac{1}{\tau }\sum_{i=(j-1)\tau +k}^{j\tau +k-1}x_{i},\;\;1\leq j\leq \frac{N}{\tau }$$
2. The complexity of the obtained coarse-grained time series can be estimated by SampEn. The number of matched vector pairs $n_{k,\tau }^{m+1}$ or $n_{k,\tau }^{m}$ is computed for each scale factor τ and all coarse-grained time series $y_{k}^{\left(\tau \right)}$. And then, the RCMSE is defined as
  (2) $$RCMSE\left(x,\tau ,m,r\right)=-ln\left(\frac{\sum_{k=1}^{\tau }n_{k,\tau }^{m+1}}{\sum_{k=1}^{\tau }n_{k,\tau }^{m}}\right)$$

RCMSE(x,τ,m,r) was calculated by using Equation (2), the undefined values in RCMSE(x,τ,m,r) exist only when all $n_{k,\tau }^{m+1}$ or $n_{k,\tau }^{m}$ are zeros. Therefore, the RCMSE algorithm can reduce the probability of undefined entropy.

## 3. Main Results

In this section, wRCMSE is constructed to identify the instability inception at different time scales. The scheme of the wRCMSE algorithm contains the obtention of statistical features for coarse-grained time series, the extraction of RCSFs, and the formation of wRCMSE, a combination of RCSFs and RCMSE.

### 3.1. Obtention of Statistical Features for Coarse-Grained Time Series

To characterize amplitude information of time series, it is supposed that *p* statistical features are chosen to specify instability inception properties. Let $X=\{x_{1},\cdots ,x_{N}\}$ represent a labeled instability time series of length *N*; six common time-domain-based statistical features (p=6) are employed in this paper, namely, skewness(SK), root mean square (RMS), kurtosis (KT), shape factor (SF), crest factor (CF), and clearance factor (CLF). The expressions for each statistical feature are widely available in [39,40].

A coarse-grained procedure defined as (1) is used to obtain the representations of the time series *X* on different time scales. For each coarse-grained time series at τ scale $y_{k}^{\left(\tau \right)}$, statistical features can be represented by a statistical feature function $S_{p,k}^{\tau }=S_{p}\left(y_{k}^{\left(\tau \right)}\right)\;\left(p=1,\cdots ,6,\;k=1,\cdots ,\tau \right)$ and can be further expressed in the form of a matrix as follows

(3) $$S_{p,k}^{\left(\tau \right)}=\left[\begin{array}{cccc} S_{1,1}^{\left(\tau \right)} & S_{2,1}^{\left(\tau \right)} & \cdots & S_{6,1}^{\left(\tau \right)}\\ \vdots & \vdots & \; & \vdots \\ S_{1,k}^{\left(\tau \right)} & S_{2,k}^{\left(\tau \right)} & \cdots & S_{6,k}^{\left(\tau \right)}\\ \vdots & \vdots & \ddots & \vdots \\ S_{1,\tau }^{\left(\tau \right)} & S_{2,\tau }^{\left(\tau \right)} & \cdots & S_{6,\tau }^{\left(\tau \right)} \end{array}\right]$$

For example, $S_{2,3}^{6}$ indicates the second time-domain features (based on $S_{2}$ operation) for the 6th coarse-grained vector series of the 3rd vector.

For instability time series *X*, the feature set D under various operating conditions can be constructed by a variety of different scales of statistical features $S_{p,k}^{\left(\tau \right)}$, where $D=[D_{1},\cdots ,D_{p},\cdots ,D_{6}]$ and $D_{p}$ is a vector representing all time scales of the *p*th statistical feature $S_{p,:}^{\left(\tau \right)}$ under various operating conditions.

### 3.2. Extraction of RCSFs

To ensure that the useful or relevant time-domain features are selected, feature selection approach is required to extract the dominant statistical features from the common time-domain statistical features. The excellent random forest is chosen because of its good accuracy, robustness, and ease of use [41]. The random forest implemented mean decrease accuracy algorithm can compute the feature importance on permuted out-of-bag (OOB) samples based on a mean decrease in the accuracy. For the normalized feature set D, the procedures for the extraction of dominant statistical features are as follows:

1. Train forest and measure OOB errors; trees in a forest can be constructed from a bootstrap sample drawn from the normalized feature set D. For each tree t,1≤t≤ntree, the prediction error $E_{tp}$ on the test data known as the OOB data are recorded.
2. Permute the *p*th feature $D_{p},\;p=1,2,\cdots ,6$ and repeat step 1, for each tree t,1≤t≤ntree, the prediction error $EP_{tp}$ on the OOB data are obtained.
3. Calculate the importance of the *p*th feature $D_{p}$, the importance indexes $I_{p}$ of the feature $D_{p}$ are defined by
  (4) $$I_{p}=\frac{1}{ntree}\sum_{t=1}^{ntree}\left(EP_{tp}-E_{tp}\right)$$
  where ntree denotes the number of trees in the forest, $E_{tp}$ denotes the OOB error on tree *t* before permuting the values of $D_{p}$, $EP_{tp}$ denotes the OOB error on tree *t* after permuting the values of $D_{p}$.

Based on the calculated feature importance, the dominant statistical features can be determined by selecting importance indexes $I_{p}$ that exceed an appropriate threshold $I_{0}$. If the threshold $I_{0}$ is determined, the statistical features corresponding to $I_{p}\geq I_{0}$ are determined as the dominant statistical features.

### 3.3. Formation of wRCMSE

**Figure 1:**
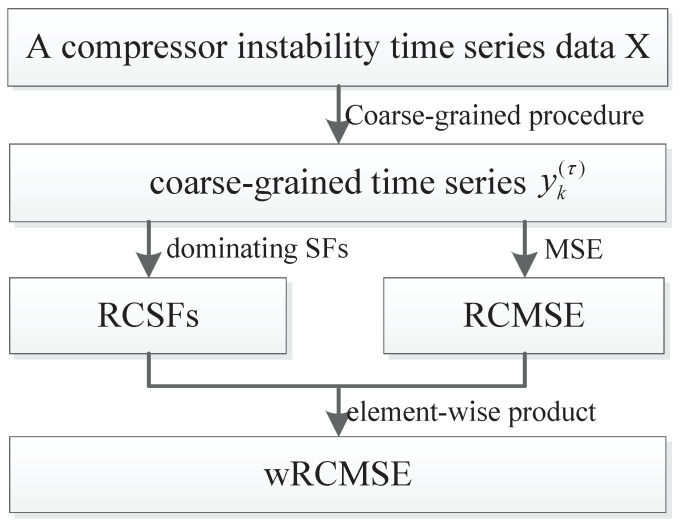
The scheme of the wRCMSE algorithm (wRCMSE is constructed by combining the dominant statistical features and RCMSE at each scale).

The wRCMSE algorithm can be constructed based on the dominant statistical features and RCMSE. The scheme and the pseudocode of the wRCMSE algorithm are shown in Figure 1 and Algorithm 1. The combined multiscale features are derived through the multiplication of the statistical and nonlinear features at each scale.

Considering the compressor instability time series *X*, first, different scales or resolutions of time series *X* can be calculated by the refined composite coarse-grained procedure defined as (1), and coarse-grained time series $y_{k}^{\left(\tau \right)}$ are obtained to represent the dynamics of the compressor instability at τ scale. Second, amplitude features of coarse-grained time series at τ scale $y_{k}^{\left(\tau \right)}$ called RCSFs can be further extracted by the dominant statistical features obtained by random forest. Since the importance indexes $I_{p}$ represent the degree of impact that *p*th statistical features generate in response to different operating conditions, the RCSFs are constructed as the sum of the dominant statistical features weighted by the normalized importance indexes and can be expressed as $w_{s}^{\tau }=\sum_{p=1}^{s}\left(I_{p}\times {\bar{S}}_{p}^{\tau }\right)$, where $w_{s}^{\tau }$ is the amplitude feature of coarse-grained time series considering *s* dominant statistical features at τ time scale, ${\bar{S}}_{p}^{\tau }=\frac{1}{\tau }\sum_{k=1}^{\tau }S_{p,k}^{\left(\tau \right)}$ is the average of the *p*th statistical feature at τ time scale. Meanwhile, the complexity of coarse-grained time series at τ scale $y_{k}^{\left(\tau \right)}$, $RCMSE(y_{k}^{\left(\tau \right)},\tau ,m,r)$, is also extracted by Formula (2). Finally, at each time scale, the newly constructed RCSFs are used as the weighted parameters and are further multiplied (element-wise) with RCMSE to generate the wRCMSE.

Based on the selection of the dominant statistical features, the wRCMSE algorithm can take different forms. Suppose that the dominant statistical feature is only RMS; RMS is described by the expression: $RMS\left(X\right)=\sqrt{\frac{1}{N}\sum_{i=1}^{N}x_{i}^{2}}$, and the importance index $I_{1}=1$. The refined composite RMS (RCRMS) at the time scale τ can be derived by using RMS to process coarse-grained time series at τ scale $y_{k}^{\left(\tau \right)}$. Therefore, RCSFs are RCRMS and are expressed as

(5) $$S_{1}^{\left(\tau \right)}=\sqrt{\frac{1}{\tau (N-\tau +1)}\sum_{k=1}^{\tau }\sum_{i=1}^{N-\tau +1}{\left(y_{ki}^{\tau }\right)}^{2}}$$

With the combination of RCRMS and RCMSE, the value of wRCMSE(X,τ,m,r) is the multiplication of these two values at each time scale:

(6) $$wRCMSE\left(X,\tau ,m,r\right)=S_{1}^{\left(\tau \right)}\times RCMSE\left(X,\tau ,m,r\right)$$

**Algorithm 1** The wRCMSE algorithm for the identification of the short-time instability inception.**Input:** The labeled instability time series, *X*, including the process of the compressor instability from normal to inception **Output:** A combined feature wRCMSE(X,τ,m,r) to distinguish the instability inception state from the health state of instability time series
1:Calculate coarse-grained time series $y_{k}^{\left(\tau \right)}$ by Formula (1);2:Construct the feature set D under the normal and instability inception operating conditions by $S_{p,k}^{\left(\tau \right)}$;3:Calculate the importance $I_{p}$ of the pth feature based on random forests;4:Set a feature importance threshold $I_{0}$, the statistical features corresponding to $I_{p}\geq I_{0}$ are the dominant statistical features;5:Determine the RCSFs by calculating the dominating statistical features on the $y_{k}^{\tau }$ at the time scale τ;6:Determine the RCMSE by calculating the MSE on the $y_{k}^{\tau }$ with the selected embedding dimension *m* and the tolerance *r*;7:Determine wRCMSE(X,τ,m,r) by the multiplication of RCSFs and RCMSE;8:**Return** wRCMSE(X,τ,m,r)


## 4. Simulations

In this section, to verify the effectiveness of the proposed wRCMSE algorithm, two synthetic noise data and a nonlinear model simulation data are analyzed with MSE, RCMSE, and wRCMSE, respectively.

### 4.1. The Complexity of Two Synthetic Noise Data

White Gaussian noise (WGN) and 1/f noise are two important signals to evaluate the multiscale entropy-based approaches. The advantages of the proposed wRCMSE algorithm in complexity measurement are illustrated through the analysis of two synthetic noise data.

**Figure 2:**
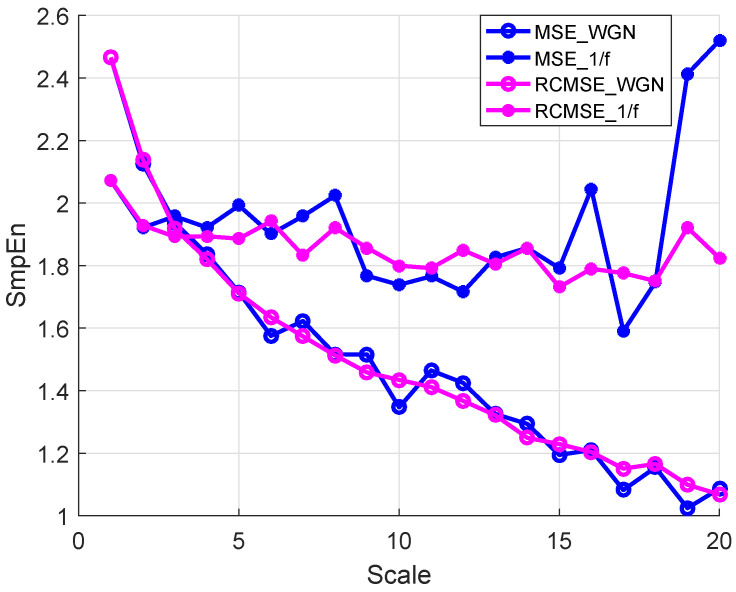
The multiscale entropy values of 1/f and WGN with 2k data points.

**Figure 3:**
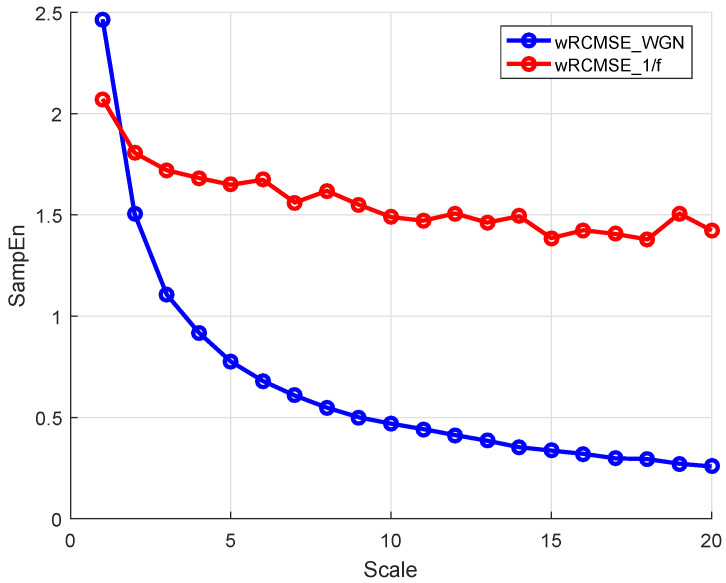
The entropy values of 1/f and WGN with 2k data points.

**Figure 4:**
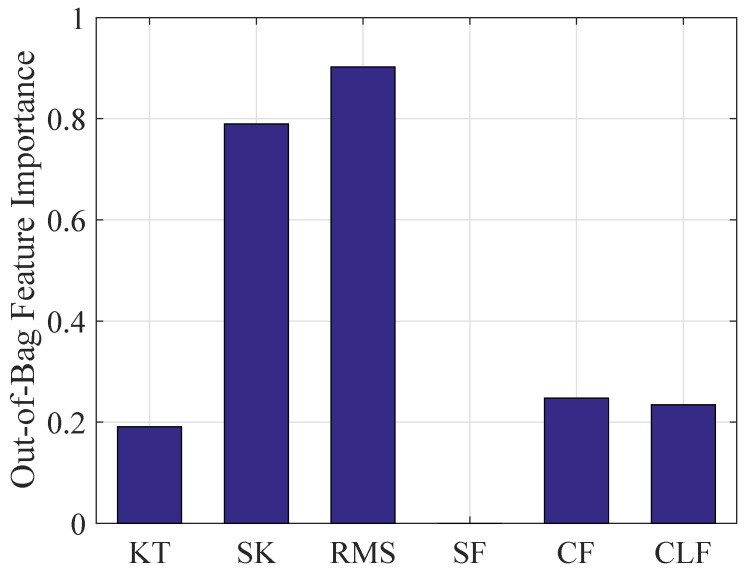
The importance of the features based on mean decreased accuracy.

For short time series, the complexity of 1/f and WGN with 2k data points are first measured with MSE and RCMSE as a comparison. The multiscale entropy values with the parameters m=2 and r=0.15δ are calculated at a time scale of 20 as shown in Figure 2. The complexity of 1/f is lower than WGN in the first three time scales, while this result changed with the increase of the time scale. With the same parameters, similar results were obtained in [33,42]. The entropy values obtained using the MSE algorithm are volatile obviously between 1.6 and 2.6, while the entropy values obtained using the RCMSE algorithm are gradually consistent with the increase of scale. Therefore, the RCMSE algorithm is superior to the MSE algorithm in the consistency of entropy estimation. Based on this advantage of RCMSE, feature values of 1/f and WGN are calculated by wRCMSE, and the results are shown in Figure 3. The change trend of features extracted by wRCMSE is consistent with that of entropy estimated by RCMSE in multiple time scales. The importance of the features can be calculated by random forests with mean decrease accuracy index and are shown in Figure 4. Considering that the correlation of features cannot be reflected in this feature selection algorithm, even if RMS and SK have a large OOB feature importance, a larger threshold should be selected, and RMS is selected as the dominant statistical feature with $I_{0}=0.8$. The result in Figure 3 shows that feature values of 1/f are greater than that of WGN at all scales except the first time scale. The feature values of WGN decrease rapidly at the initial several time scales and are gradually consistent with the increase of time scale, while the feature values of 1/f tend to be constant for all scales. Although the proposed feature extraction approach includes statistical features, it can still measure the complexity of time series because the statistical features can be regarded as weighting factors. Therefore, the wRCMSE algorithm maintains the advantages of the RCMSE algorithm and can further improve the measurement accuracy of entropy.

### 4.2. The Identification of the Instability Inception

#### 4.2.1. The Instability Data Obtained by the Mansoux Model

An excellent instability model called the Mansoux model [13] is used to generate the instability simulation data. The Mansoux model could describe the transient behavior of instability inception and coincide well with experimental results within a certain precision [43].

The Mansoux model is expressed in the form of the following state-space equations:

(7) $$\left\{\begin{array}{c} E\dot{\phi }=-A\phi +\psi_{c}\left(\phi \right)-T\psi_{p}\\ \dot{\psi_{p}}=1/\left(4l_{c}B^{2}\right)\left(S\phi -\Phi_{T}\left(\psi_{p}\right)\right) \end{array}\right.$$

where ϕ and $\psi_{p}$ are the non-dimensional flow coefficient and the plenum pressure, respectively. $\psi_{c}$ is an important nonlinear function, which represents the characteristic function of a compressor. $\Phi_{T}\left(\psi_{p}\right)=\gamma \sqrt{\psi_{p}}$, $\gamma =\sqrt{2/K_{T}}$, $K_{T}$ is the control parameter related to the degree of throttle closure. The specific form of matrixes (*E* and *A*), the vector (*S* and *T*), and the scalar ($l_{c}$ and *B*) can be found in [13,43].

**Figure 5:**
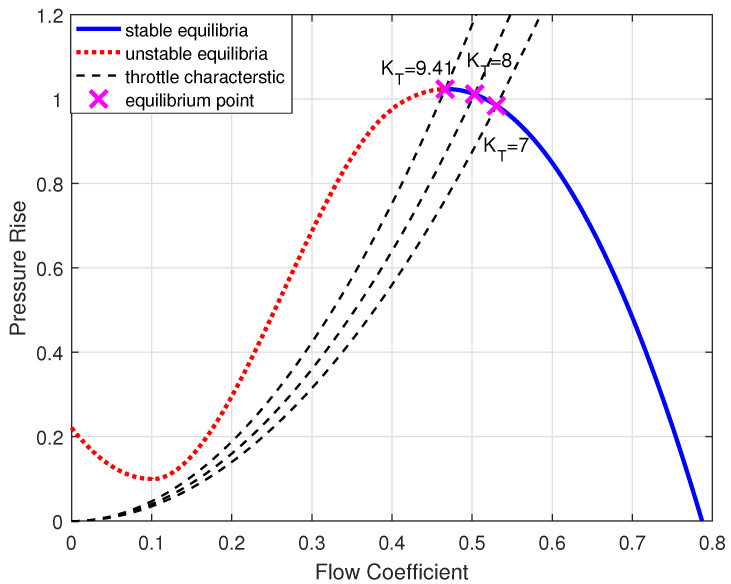
Compressor and throttle characteristics used to simulate instability in a C2 compressor.

The compressor instability process is simulated by continuously adjusting the Mansoux model parameter $K_{T}$ from 7 to 9.41. The parameter $K_{T}$ adjustment represents the gradual closure process of the throttle in the compressor surge experiment. At stall inception, $K_{T}$ is 9.41 [13]. The axisymmetric compressor characteristic $\psi_{c}$ is as follows, and the corresponding characteristic shapes are illustrated in Figure 5.

(8) $$\psi_{c}=\left\{\begin{array}{c} 12.117\phi^{2}-2.423\phi +0.221;\;\phi <0.1\\ -49.624\phi^{3}+39.509\phi^{2}-6.413\phi +0.395;\;0.1\leq \phi \leq 0.4\\ -10.0695\phi^{3}+9.430\phi -1.184;\;\phi >0.4 \end{array}\right.$$

The stability of the equilibrium point is represented by the intersection between $\psi_{c}\left(\phi \right)$ and $\psi_{T}\left(\phi \right)$. Instability occurs at the point where the slope of the characteristic curve is zero.

**Figure 6:**
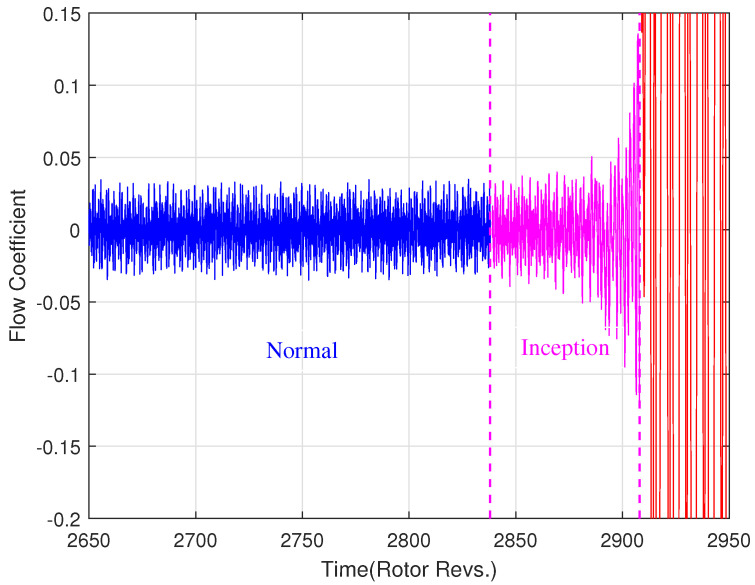
A detrended flow coefficient ϕ is divided into three states: normal (the blue line), instability inception (the magenta line), and instability (the red line).

A flow coefficient ϕ evolution from normal to instability can be calculated and is shown in Figure 6. The flow coefficient includes 3000 rotor revolutions (60k data points with 0.05 time interval in the simulation). In data preprocessing, the Savitzky-Golay filter is implemented to eliminate the trend and WGN with 0.4 times the maximum amplitude of the first 10k data is added to verify the robustness of the wRCMSE algorithm. The developing process of instability could be divided into three states: normal (the blue line), instability inception (the magenta line), and instability (the red line). In Figure 6, the time intervals corresponding to the three states are [0,2817], [2818,2908], and [2909,3000], respectively. The instability inception contains a small amount of data with 1800 data points (90 rotor revolutions) and its detection could be used as an early warning sign to further prevent compressor instability. To obtain an accurate identification, 20 samples with different time intervals from 40 to 85 rotor revolutions are selected to extract the features of the instability inception. Only four different time intervals 500, 1000, 1500, and 2000 rotor revolutions are selected as the normal samples, because there are sufficient data lengths to feature extraction in normal operating conditions.

#### 4.2.2. Selection of the Parameters

The calculation of wRCMSE requires a priori specification of parameters: the embedding dimension *m*, a tolerance threshold *r*, and the dominant statistical features. The parameter *m* determines the length of the sequences to be compared, and its selection can be estimated by calculating the false nearest neighbor. The second parameter, *r*, is the tolerance threshold for accepting similar patterns between two segments.

To show the influence of embedding dimension *m* on the calculation of SampEn, normal data with sufficient data length are analyzed with different *m* values. SampEn(ϕ, m, 0.15σ, 10,000) and SampEn(ϕ, m, 0.2σ, 10,000) of the four normal samples are calculated with m=2,3, and 4, and then the mean, the standard deviation (SD) and the coefficient of variation (CV) of SampEn are shown in Table 1 and Table 2, respectively. The higher CV shows a bigger SD and a wider spread of SampEn. When r=0.15σ and the sample length N = 10,000, the SD and CV of SampEn(ϕ, m, 0.15σ, 10,000) exponentially increase with the embedding dimension *m*. Similar results are also obtained by SampEn(ϕ, m, 0.2σ, 10,000) with r=0.2σ and N = 10,000. Therefore, the specific value of m=2 is selected for the minimum SD and CV of SampEn.

**Figure 7:**
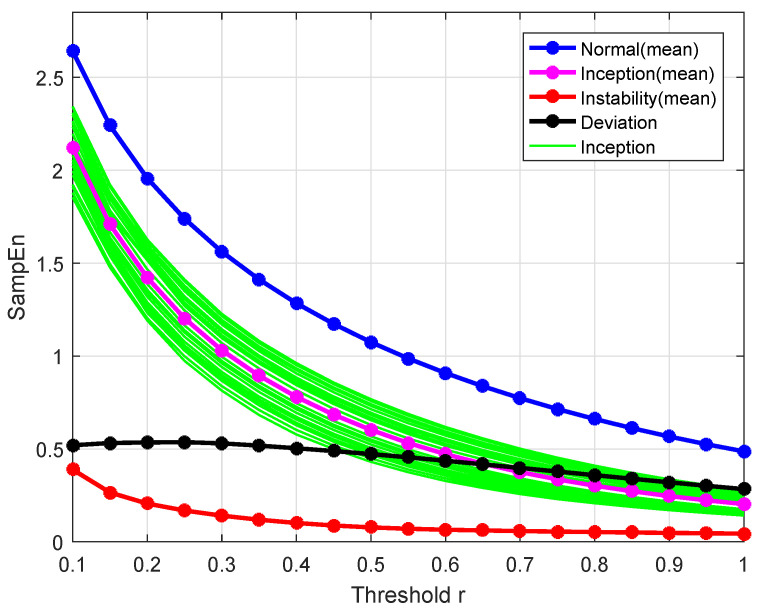
SampEn of the simulation data under different operations and tolerances.

According to the selection of *r* in ApEn [32], there are two different approaches to determine the tolerance *r*, one using the deviation of SampEn for both normal and instability inception conditions, the other is the calculation of the mean SampEn at different tolerances. Calculations of the SampEn of each state and the mean of SampEn in each class are shown in Figure 7. As the tolerance *r* increases, the means of SampEn for both normal and instability inception conditions gradually decrease and are represented by the blue and magenta lines, respectively. The deviation between these two means is represented by the black line, and the peak of the deviation (r=0.25σ) can be selected as the appropriate tolerance *r*. In addition, the means of SampEn for stall conditions showed by the red line tends to constant value gradually when r>0.5σ. It is appropriate that the tolerance *r* is selected in the range of 0 to 1.0σ. Another approach is employed for the analysis of an unknown sample without any information available. The mean SampEns for both normal and instability inception conditions are calculated as 1.5396 and 1.0867 at four different tolerances including r=0.1σ,0.2σ,0.5σ, and 1.0σ. SampEn(ϕ,2,0.25σ, 10,000) for both normal and instability inception conditions are 1.7390 and 1.2025 and close to the mean SampEns. Therefore, the parameters m=2 and r=0.25σ are selected in the wRCMSE algorithm.

**Figure 8:**
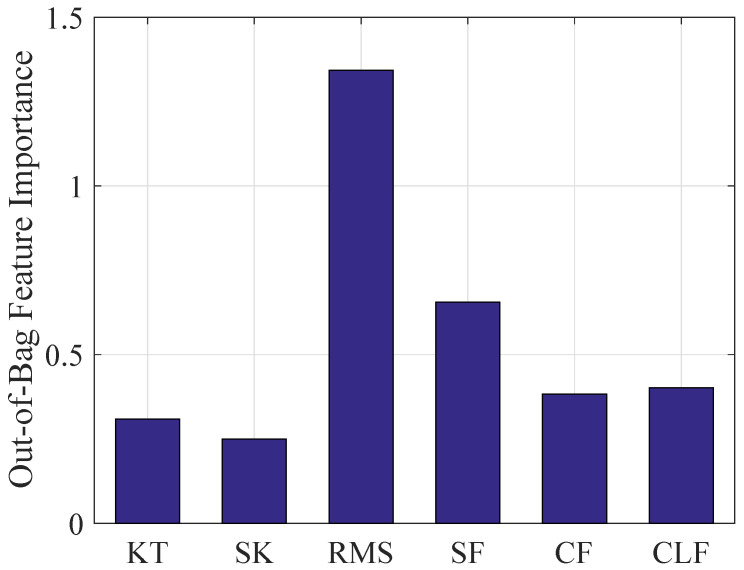
The importance of the features based on mean decreased accuracy.

In addition, the same six time-domain features are used to specify instability inception properties, and the importance of the features can be calculated by random forests with mean decrease accuracy index and are shown in Figure 8. This result shows that RMS has the largest OOB feature importance while the importance of the other five features is relatively small and close. The selection threshold is $I_{0}=1$ and RMS is used as the dominant statistical features.

#### 4.2.3. The Validity and Robusticity of wRCMSE

**Figure 9:**
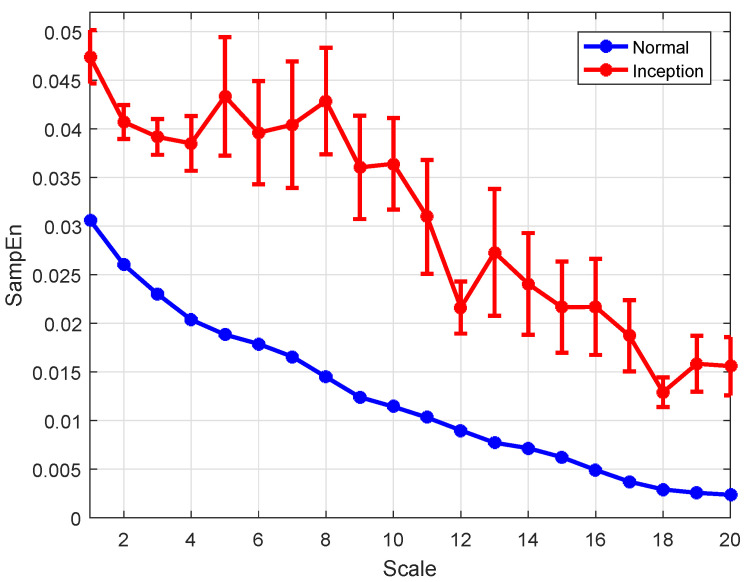
The features of the normal and the instability inception using wRCMSE (values are given as means ± standard error).

The features of the normal and the instability inception at scales 1 to 20 are calculated using the wRCMSE algorithm, the results are given as the mean value of entropy ± standard error and are shown in Figure 9. The features of the normal gradually decrease as the time scale increases while the features of the instability inception fluctuate greatly with the increase of the time scale. The features of the normal are all lower than those of the instability inception at each scale and this result indicates that these two operating conditions can be clearly distinguished by these features.

**Figure 10:**
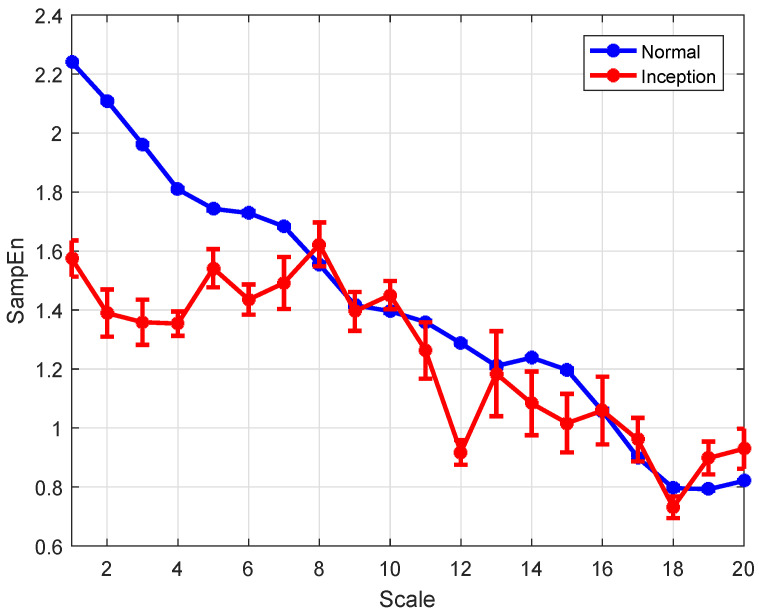
The entropies of the normal and the instability inception using RCMSE (values are given as means ± standard error).

In Figure 9, the features calculated by the wRCMSE algorithm are well-defined for all samples with different time scales and different data lengths. By comparison, the entropies are also calculated by the MSE and a modification of the MSE (wMSE) algorithm. Based on a weighted approach in [44], wMSE can be constructed to incorporate significant information from the time series when computing the matched vector pairs. The undefined entropies calculated by the MSE and wMSE occur with the time scale increased. The undefined entropies are distributed mainly at scales 15, 18, 19, and 20, and the probability of the undefined entropies is 3% when the MSE and wMSE algorithms are applied to analyze the instability inception. Regarding validity, the wRCMSE algorithm is superior to both MSE and wMSE algorithms for the short time series. Moreover, the entropies of the normal and the instability inception at scales 1 to 20 are calculated using RCMSE and are shown in Figure 10, the entropies of the normal are greater than that of the instability inception at the first seven scales while these entropies are almost equal at the latter scales. Although these two operating conditions can be clearly distinguished by the entropies at the first few scales, it is considered unreasonable that the entropy of the normal one be larger than that of the instability inception. In general, the development of the instability is a nonlinear process and the instability inception stage is associated with the emergence of the more nonlinear behavior. The instability inception has an increase in dynamical complexity and the entropies of the instability inception are greater than those of the normal.

**Figure 11:**
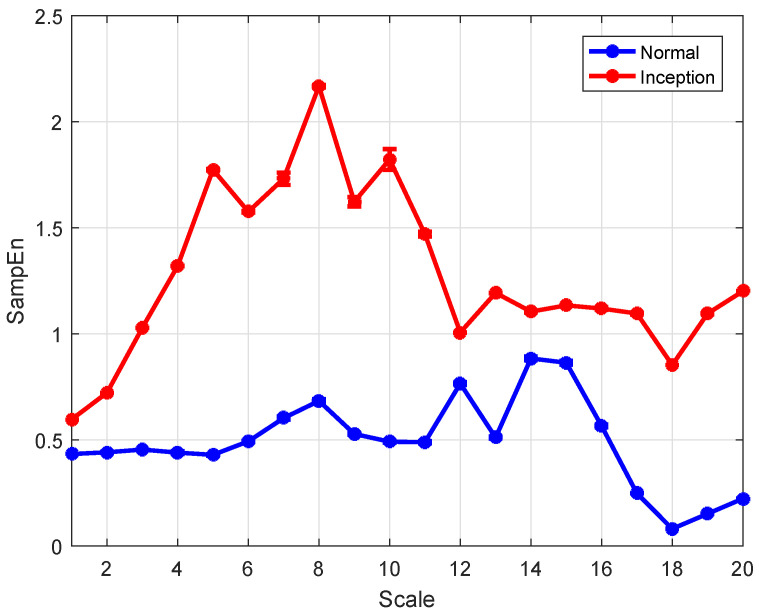
The entropies of the normal and the instability inception without random hit noise using RCMSE.

**Figure 12:**
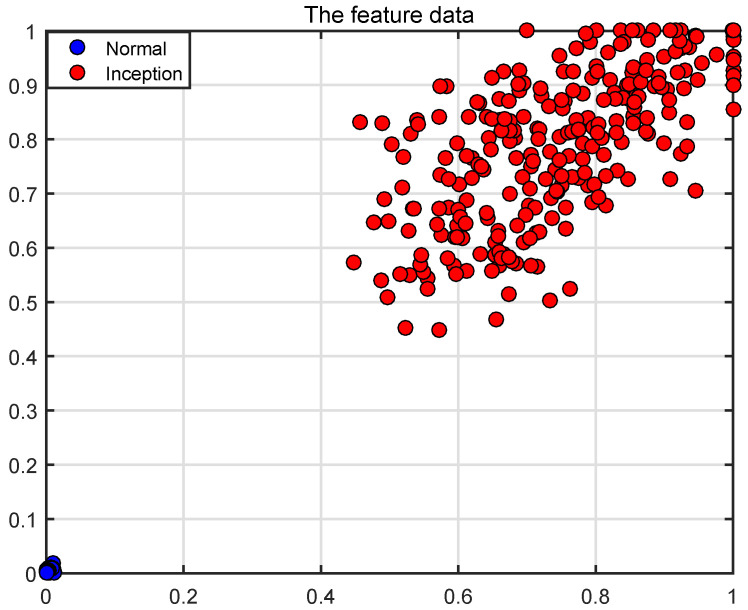
The feature distribution of the flow coefficient under WGN with different amplitudes.

In Figure 9, the features of the instability inception are greater than those of the normal at all scales. The features calculated by wRCMSE can retain the change trend of multiscale entropies calculated by RCMSE and avoid the unreasonable result in Figure 10. These two operating conditions can be clearly distinguished by these features; therefore, the instability inception can be detected by a simple classifier. The features of the normal have similar tendencies to the entropies of WGN in Figure 2. It happened because the normal and the instability inception has been submerged in noise, when the flow coefficient of a WGN is added to the flow coefficient. The comparison results of the two operating conditions are consistent with those without WGN in Figure 11. In addition, the flow coefficient under WGN with different amplitudes (0.2 and 0.3 times the state amplitude under the normal operations) are also analyzed by the wRCMSE algorithm and the same results are obtained. These results show the wRCMSE algorithm with high robustness against the outside disturbance. Considering the flow coefficient under WGN with different amplitudes, the entropies of the flow coefficient at all scales can be divided into two-dimensional data through the combination of two-scale data. The distribution of the two-dimensional data is shown in Figure 12. The nonlinear feature distribution can be easily classified by the support vector machine and the accurate classification of the instability inception can be obtained with the early detection of the compressor instability.

## 5. Conclusions

In this paper, a combination of RCSFs and RCMSE approach wRCMSE was proposed to identify the short-time instability inception. The multiscale statistical features and multiscale nonlinear features of the instability inception can be extracted by wRCMSE to distinguish the instability inception state from the health state of the instability time series. Two synthetic noise data and a compressor instability model data were used to evaluate the validity and robusticity of wRCMSE. Simulation results revealed that the wRCMSE algorithm could inherit the advantages of the RCMSE algorithm and achieve robustness to external disturbance. The multiscale features calculated by wRCMSE retained the change trend of multiscale entropies calculated by RCMSE and avoided the inaccurate measurements or undefined entropies of MSE and wMSE for a short time series. In addition, the instability inception under WGN with different amplitudes could be measured and identified by wRCMSE for each of the scales or resolutions, and the instability inception could be clearly distinguished from the health state of axial compressors at all scales. The early detection of the compressor instability could be further achieved to classify the instability inception by the traditional support vector machine algorithm.

## Figures and Tables

**Table 1:** Statistic values of SampEn(ϕ, *m*, 0.15σ, 10,000) of the normal samples when the parameters r=0.15σ and *m* is 2,3 and 4.

*m*	*m* = 2	*m* = 3	*m* = 4
Mean	2.2426	2.2386	2.2451
SD	0.0066	0.0157	0.0381
CV	0.0029 ^1^	0.007	0.017

^1^ the lowest CV value at m=2.

**Table 2:** Statistic values of SampEn(ϕ, *m*, 0.2σ, 10,000) of the normal samples when the parameters r=0.2σ and *m* is 2,3 and 4.

*m*	*m* = 2	*m* = 3	*m* = 4
Mean	1.959	1.966	1.9633
STD	0.0069	0.0098	0.0188
CV	0.0035 ^2^	0.0049	0.0096

^2^ the lowest CV value at m=2.

## Data Availability

The data that support the findings of this study are available from the corresponding author upon reasonable request.

## Abbreviations

The following abbreviations are used in this manuscript:

MSE	Multiscale entropy
ApEn	Approximate entropy
SampEn	Sample entropy
RCMSE	Refined composite multiscale entropy
RCSFs	Refined composite statistical features
wRCMSE	A combination of RCSFs and RCMSE
RMS	Root mean square
HMRDE	Hierarchical multiscale reserve dispersion entropy
